# Study on fold formation mechanism and process optimization in multi-directional die forged valve bodies

**DOI:** 10.1371/journal.pone.0337844

**Published:** 2025-12-26

**Authors:** Longjiang Niu, Yongwan Zhang, Leiyu Zhang, Qingliang Zhang, Weiping Luo, Jingyu Wang, Donghang Liu, Babak Ziaie, Xavier Velay

**Affiliations:** 1 School of Mechanical Engineering, Shanghai Dianji University, Shanghai, China; 2 Jiangsu Zhonggong New Energy Technology Co., Ltd., Rugao City, Nantong, Jiangsu Province, China; 3 Faculty of Engineering and Computing, Atlantic Technological University, Sligo, Ireland; IGDTUW: Indira Gandhi Delhi Technical University for Women, INDIA

## Abstract

Fold defects represent a prevalent and detrimental issue in the multi-directional die forging of complex valve bodies, often resulting in product rejection and increased manufacturing costs. In this study, a three-dimensional thermo-mechanical coupled finite element (FE) model was developed using Forge^®^ software to simulate the multi-directional die forging process. The “marking grid” and “sensors” functionalities were employed to visualize and track the formation and evolution of fold defects throughout the entire forming process, thereby elucidating the underlying fold formation mechanism. The effects of three key process parameters—initial billet temperature, main punch speed, and friction coefficient between the billet and die—on fold depth and damage value were systematically analyzed. Orthogonal experiments combined with analysis of variance (ANOVA) were conducted to identify the optimal parameter combination. Results indicated that the friction coefficient had the most significant influence on fold formation and damage accumulation, followed by billet temperature, while punch speed had the least impact. The optimal parameters were determined to be a friction coefficient of 0.1, an initial billet temperature of 1200 °C, and a main punch speed of 30 mm/s. Production trials and fluorescent magnetic particle inspection confirmed the absence of fold and crack defects, and the final forged product closely matched the simulation predictions, validating the effectiveness of the optimized process.

## 1. Introduction

Multi-directional die forging technology has emerged as a pivotal near-net shape forming method to produce valve bodies with multiple cavities, which has wide application in marine, aerospace, petrochemical and power industries [1,2].

A large body of scholarly work has been dedicated to the investigation of multi-directional die forged valve bodies. Gontarz [1] compared two alternative forging processes for the fabrication of valve bodies with three equal port diameters, evaluating them in terms of stress state index, tool work input, and strain distribution. The findings highlighted that the three-slide forging press (TSFP) is the most recommended method for such forging operations. Cao et al. [2] investigated a multi-directional forged multi-cavity part, showing that when length of the main pipe equals to its outer diameter or the inner-outer diameter ratio of main pipe ≤ 0.6, fold presented in the inner wall of the main pipe. Xu [3] employed elasto-plastic FE simulation combined with experimental validation to derive the general laws governing metal flow during the multi-directional die forging of valve bodies with three equal port diameters. Sun et al. [4] focused on an AISI-5140 steel equal-diameter triple valve body, exploring the effects of key process parameters on the multi-way loading forming process, as well as the inhomogeneity of deformation and temperature distribution. Yin et al. [5] utilized Deform-3D to simulate the forging processes of a copper alloy valve body on both a conventional double-action press and a multi-ram press. Their results revealed that the multi-ram forging process effectively prevents cold shut defects in the central valve hole and enhances the overall quality of the valve body. Ji et al. [6] adopted Deform software to simulate the multi-ram die forging process of a cutoff valve and employed response surface methodology (RSM) to optimize critical parameters including forging temperature, punch speed, and friction coefficient. Zhang et al. [7] developed an FE model for the multi-ram forged cross valve within the Deform-3D environment, emphasizing the crucial role of thermal events in the forming process of aluminum alloys under multi-way loading conditions. Zhang et al. [8] conducted 2D FE simulations to study fold defects on the inner surface of inner-grooved copper tubes during ball spin forming and proposed effective preventive measures. Chan et al. [9] systematically investigated the mechanism of fold formation in asymmetrically flanged parts via FE simulation and established a systematic design framework for predicting and avoiding such fold defects. Gao et al. [10] developed a versatile approach for predicting three typical types of fold defects, which integrates a fold index, judgment criteria, and a mathematical model correlating the fold index with forming parameters. Li et al. [11] combined FE simulation and orthogonal experimental design to analyze and predict the crack initiation behavior of three-way valve forgings, concluding that cracks are more likely to occur with a decrease in die radius or an increase in friction coefficient. Most recently, Yang et al. [12] studied the forging process of C83600 tin bronze valve bodies based on rheological behavior and hot processing diagrams, successfully producing valve bodies free from defects such as folds.

Despite the extensive research efforts mentioned above, it is important to note that fold defects remain a predominant category of defects in forging operations, particularly in multi-directional die forging processes characterized by highly intricate metal flow dynamics [13]. However, most previous studies have centered on the deformation mechanisms, material flow patterns, strain-stress distributions, forming loads, loading paths, and microstructure evolution of multi-directional die forged valve bodies—with a primary focus on relatively simple valve body structures featuring equal port diameters. Research specifically addressing fold defects in multi-directional die forged valve bodies is relatively limited. Furthermore, the existing studies that do touch upon fold defects either rely on simplified 2D models, assume axisymmetry, or only investigate fold defects on the inner surfaces of cavities, thus failing to accurately replicate the complex 3D thermo-mechanical conditions of practical forging processes for valve bodies with unequal port diameters.

To address these limitations, the present study develops a 3D thermo-mechanical coupled FE model for the multi-directional die forging of valve bodies with unequal port diameters, using Forge^®^ software (Version 3.1, Transvalor S.A., Biot, France). By leveraging the “marking grid” and “sensors” features of Forge^®,^ the formation and evolution of 3D fold defects throughout the entire forming process are comprehensively visualized and tracked, facilitating an in-depth understanding of the fold formation mechanism. Based on the established FE model, a systematic investigation is conducted to assess the impacts of key process parameters—billet initial temperature, main punch speed, and billet-die friction coefficient—on fold defect formation and material damage values.

**Fig 1:**
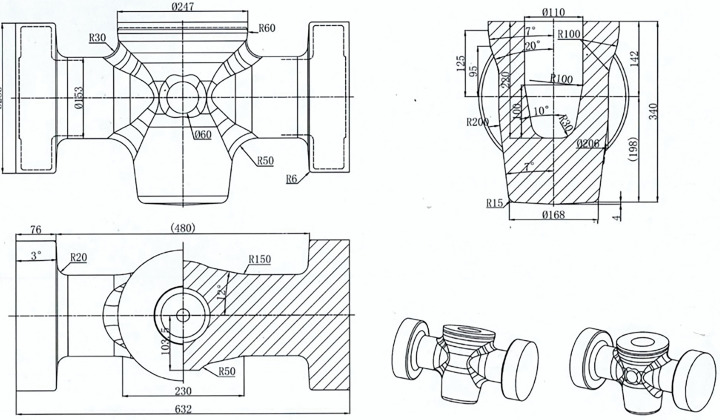
Forging drawing of the valve body.

**Fig 2:**
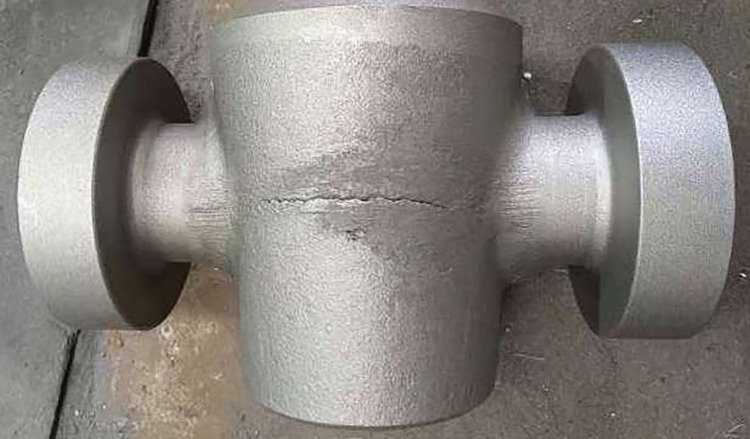
Multi-directional die forged valve body with folds.

Fig 1 illustrates the forging design of a valve body featuring ports of varying diameters, manufactured using a multi-directional die forging press. Fig 2 shows a forged valve body with a severe fold defect. Under the current unoptimized process conditions, nearly all forged parts exhibit fold defects, more than 50% of which are as severe as indicated in Fig 2, incurring substantial financial losses. Therefore, clarifying the fold formation mechanism and developing effective process optimization strategies for the multi-directional die forging of valve bodies are of critical industrial significance.

## 2. Finite element simulation of the fold-forming process

### 2.1 Constitutive equation of AISI 4130

The valve bodies investigated in this study are fabricated from AISI 4130 steel, whose chemical composition is presented in Table 1.

**Table 1:** Chemical composition of AISI 4130 (Wt%).

C	Mn	P	S	Si	Cr	Mo
0.28-0.33	0.40-0.60	0.035	0.040	0.040	0.80-1.10	0.15-0.25

The Hansel–Spittel constitutive equation is selected for modeling the thermo-viscoplastic behavior of AISI 4130 steel, owing to its balanced combination of theoretical rigor and practical applicability. This equation effectively captures the effects of temperature, strain, and strain rate—including work hardening and dynamic softening phenomena—while maintaining sufficient simplicity for widespread implementation in FE simulation software such as Forge^®^ [14–18]. The Hansel–Spittel thermo-viscoplastic constitutive model employed in this study is expressed as Equation (1):


$$\sigma =A\cdot e^{m_{\text{1}}\cdot T}\cdot \varepsilon^{m_{\text{2}}}\cdot {\dot{\varepsilon }}^{m_{\text{3}}}\cdot e^{\frac{m_{\text{4}}}{\varepsilon }}$$ (1)

where σ denotes stress, ε- strain, $\dot{\varepsilon }$-strain rate, T-temperature and A, $m_{\text{1}}$, $m_{\text{2}}$, $m_{\text{3}}$, $m_{\text{4}}$ are constants whose values, obtained from relevant literature [19], are presented in Table 2.

**Table 2:** Values for constants in the Equation (1).

𝐀	${𝐦}_{1}$	${𝐦}_{2}$	${𝐦}_{3}$	${𝐦}_{4}$
1294.64	−0.00254	−0.05621	0.1455	−0.0324

### 2.2 Establishment of the FE model

**Fig 3:**
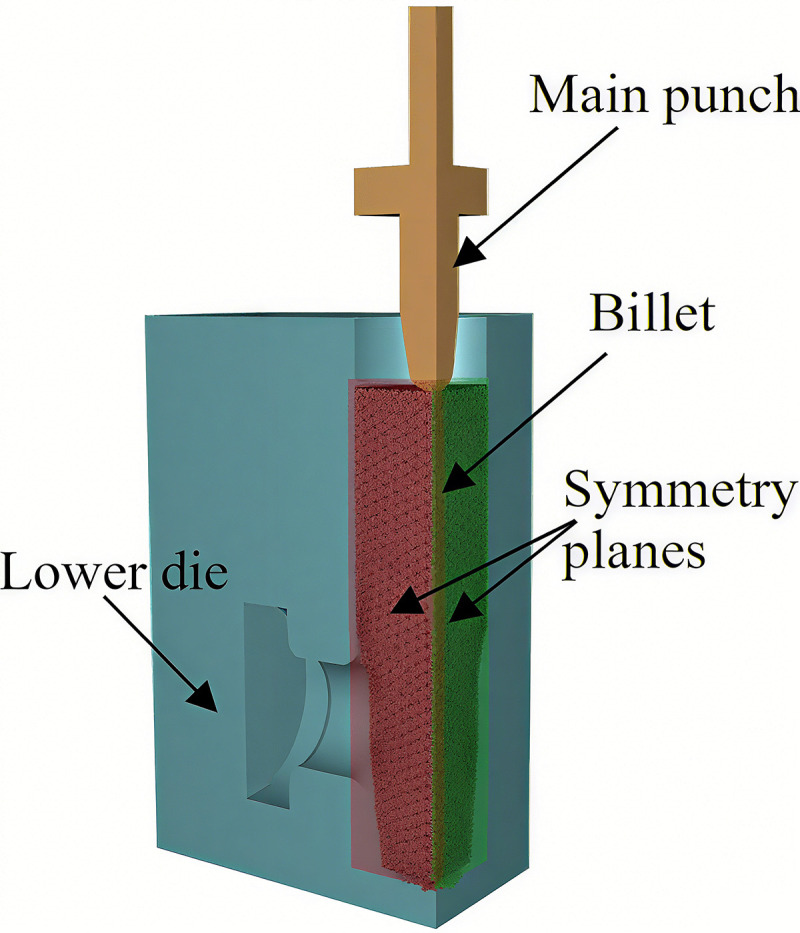
Finite element model for the forging of the valve body.

Forge^®^ is a state-of-the-art FEM-based process simulation system, widely recognized for its robustness in analyzing metal-forming and heat-treatment processes [20–22]. Fig 3 illustrates the FE model constructed for the multi-directional die forging of the studied valve body. To balance computational accuracy and efficiency, a 1/4 symmetric model of the actual valve body structure is adopted, due to the geometric symmetry of the forging. 2 symmetry planes are indicated in Fig 3.

**Fig 4:**
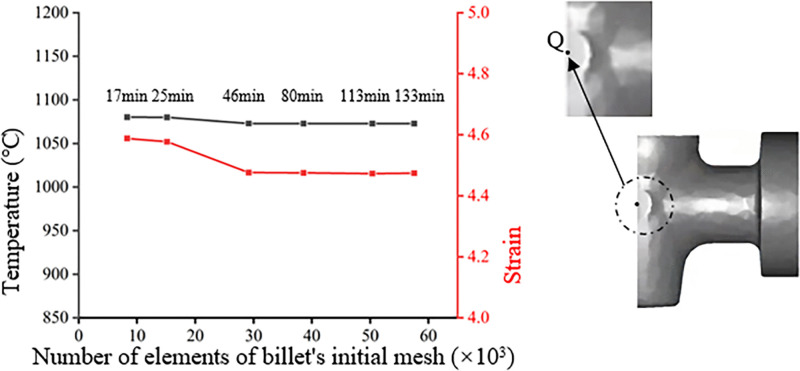
Mesh sensitivity analysis of the simulation.

A mesh sensitivity analysis is performed to determine the optimal initial mesh density for the billet, as shown in Fig 4. The analysis focuses on the temperature and equivalent strain at a critical monitoring point (Point Q, shown in the inset of Fig 4). The results reveal that when the initial number of billet elements ranges from 30,000–55,000, the variations in temperature and equivalent strain at Point Q are minimal—despite a nearly threefold increase in computational time. Consequently, an initial mesh consisting of 30,000 quadratic tetrahedral elements was selected for the billet. This mesh configuration offers distinct advantages, including enhanced solution accuracy and superior representation of curved die surfaces, which are crucial for capturing the complex metal flow.

The 3D geometric models of the components were created and assembled with NX software (Siemens AG, Munich, Germany). Each component model was then exported in the STL (StereoLithography) file format and imported into Forge^®^ for FE model construction. In the FE analysis the tools were treated as rigid bodies (i.e., non-deformable), eliminating the need to assign material properties to these parts. The key process parameters adopted in the FE simulations are summarized in Table 3. The simulation was an 8-core parallel computation, running on a workstation with 2 Intel(R) Xeon(R) Platinum 8160 CPUs and 160G of random-access memory (RAM).

**Table 3:** Process parameters.

Parameters	Value
Billet’s initial temperature	1100°C
Die’s initial temperature	250°C
Friction coefficient (billet-die interface)	0.15 (water+graphite)
Heat transfer coefficient (billet-die interface)	2000 W/m^2^·K
Heat transfer coefficient (billet-air interface)	10 W/m^2^·K
Main punch speed	20 mm/s
Number of initial billet’s element	30,000

### 2.3 Simulation of the fold evolution

**Fig 5:**
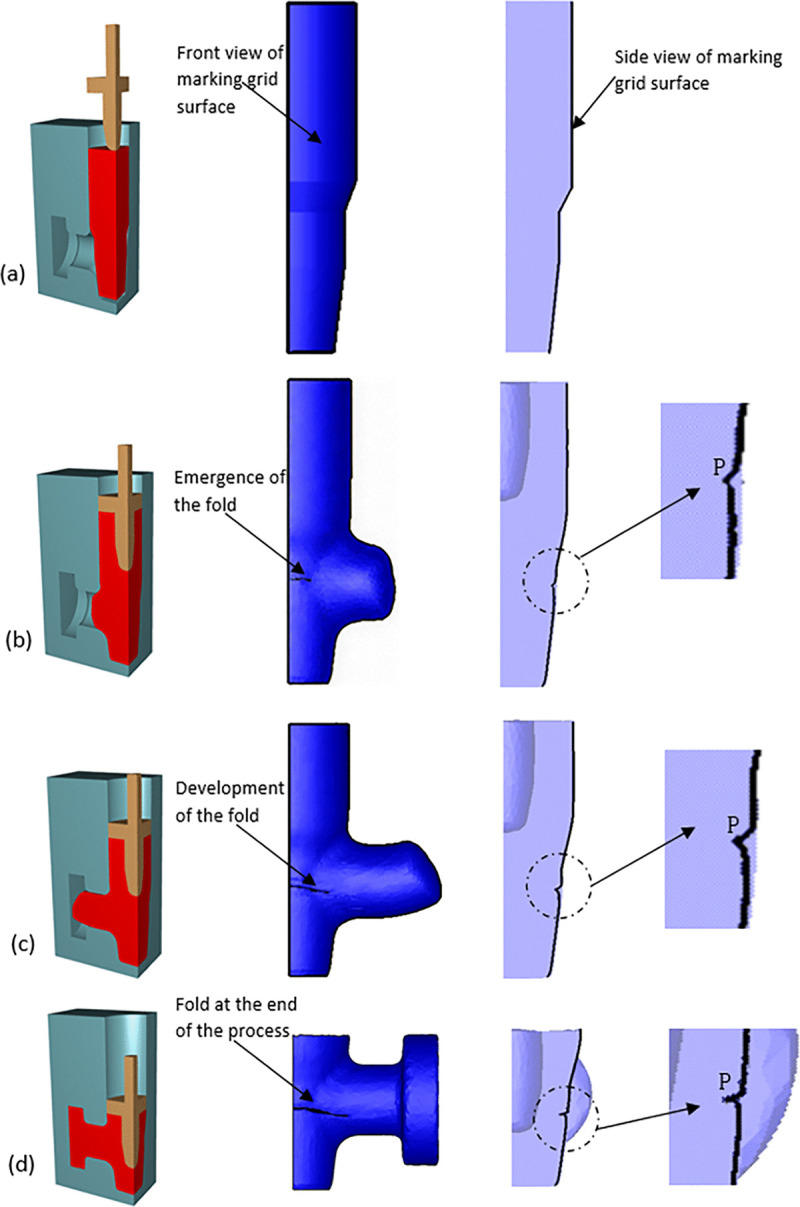
Forging process of the valve body. (A) beginning (B) 40% stroke (C) 70% stroke (D) end of stroke.

The marking grid technique is a powerful post-processing tool widely used in FE simulations to track the evolution of critical regions within the workpiece and identify defects such as folds [23]. In Fig 5 the middle column (blue surface) presents the front view of the marking grid, which visually captures the initiation and progressive development of the fold defect. The rightmost column displays the side view of the marking grid surface, represented by the black contour line superimposed on the light-purple billet model. Fig 5A–5D corresponds to the billet state at initial stage, 40% stroke completion, 70% stroke completion, and the end of the forging process, respectively, providing a clear visualization of the morphological evolution of the fold defect. At 40% stroke completion, a fold defect with a horizontal “V”-shaped cross-section emerged on the initially smooth surface of the billet. As the forging process progresses to 70% stroke completion, the “V”-shaped profile of the fold deepens, and its apex (point P, highlighted in the inset detail views) migrates further inward. By the end of the process, Point P reached its innermost position, causing the two “wings” of the fold to come into contact, resulting in a fully developed fold defect.

**Fig 6:**
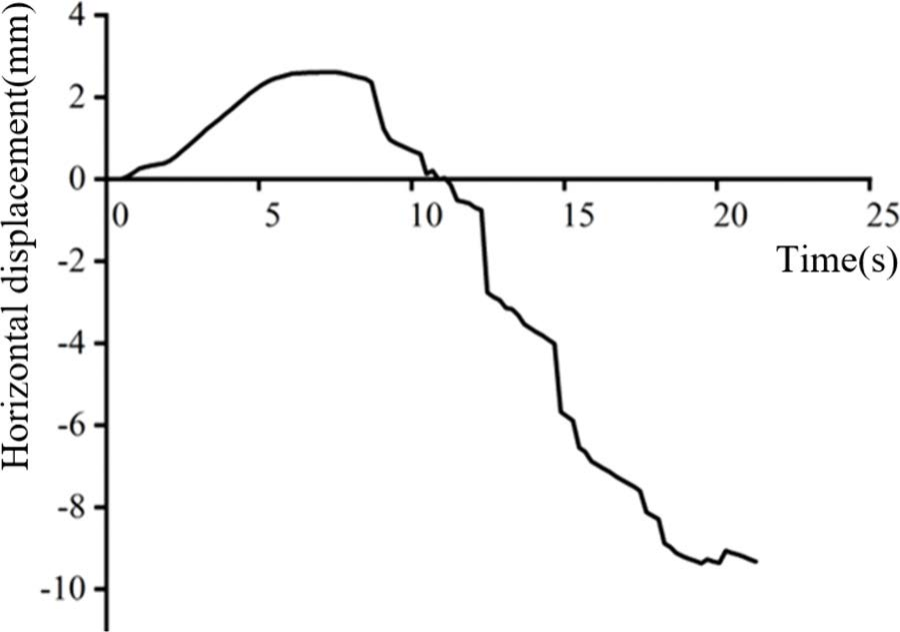
Fold depth evolution with time.

Another advanced functionality within Forge^®^’s post-processing module is the “sensors” tool, which enables real-time tracking and recording of computed variables (such as displacement, temperature) at user-defined critical points [24]. In this study, a sensor is placed at point P (the apex of the fold) to monitor its horizontal displacement history. The results, presented in Fig 6, show that in the early stage of the forging process, the gap between the billet and the die allows point P to migrate outward. Once this gap is closed due to the billet being pressed against the die wall, the horizontal displacement of Point P exhibits a linear inward trend with increasing time or stroke. At the end of the simulation, the simulated fold depth reaches approximately 10 mm.

**Fig 7:**
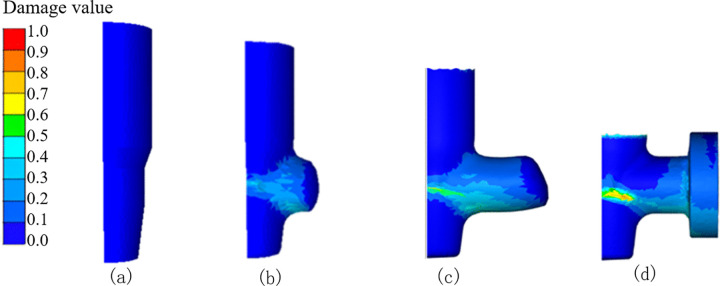
Damage values at different stages.

The corresponding material damage values at different stages of the forging process are illustrated in Fig 7. Damage values, in the context of metal forming, refer to the cumulative degradation of material integrity during deformation, reflecting the potential for ductile failure, which can be predicted and analyzed using simulation software [25]. It is a metric used to quantify the level of damage accumulated during the forging process [26,27]. As demonstrated in Figs 5 and 7, the fold depth and length increase with the advancement of the forging stroke. Importantly, the peak damage values are found to be localized precisely at the fold defect site, indicating a strong correlation between fold formation and material damage accumulation.

The FE simulation indicates that during the multi-directional die forging of the valve body, the billet’s surface material flows inwards. Due to oxidation or the scale on the billet’s surface, folds resulting from the inward material flow could even lead to crack. Oxide scale on the billet surface induces stress concentrations at fold initiation zones, where elevated damage accumulation could trigger crack formation. The spatial coincidence between fold geometry and high-damage regions confirms that folds result from coupled process parameters interactions.

## 3. Analysis of the influence of different factors on fold depth and damage values

### 3.1 Influence of the main punch speed on the fold and damage accumulation

**Fig 8:**
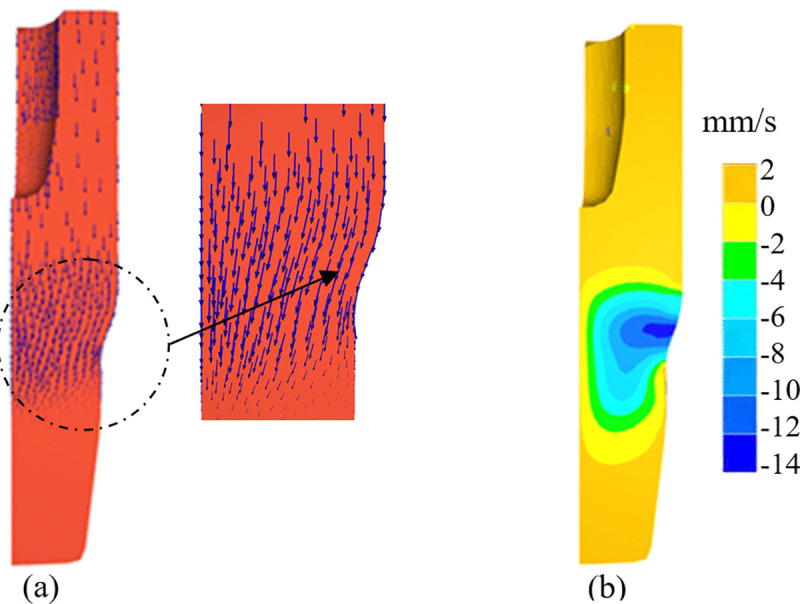
Velocity of material flow. (A) vector form (B) horizontal component of the material flow velocity.

Fig 8 illustrates the material flow velocity at one moment during the multi-directional forging of the valve body. Fig 8A presents the velocity vectors while Fig 8B displays the horizontal component of the flow speed. The studied valve body features a tapered segment as depicted in Fig 1. Within the corresponding taper section of the forged billet, as the material flows through the taper region of the die cavity, its flow direction transitioned from a predominantly vertical downward to a downward-leftward. This transition introduces a horizontal velocity component directed toward the interior of the billet. Fig 8B further demonstrates that within the taper section the closer the material is to the billet’s surface, the higher its leftward (inward) flow velocity. This specific material flow pattern promotes the inward migration of surface material, creating conditions conducive to the formation of fold defects and the accumulation of high damage values.

**Fig 9:**
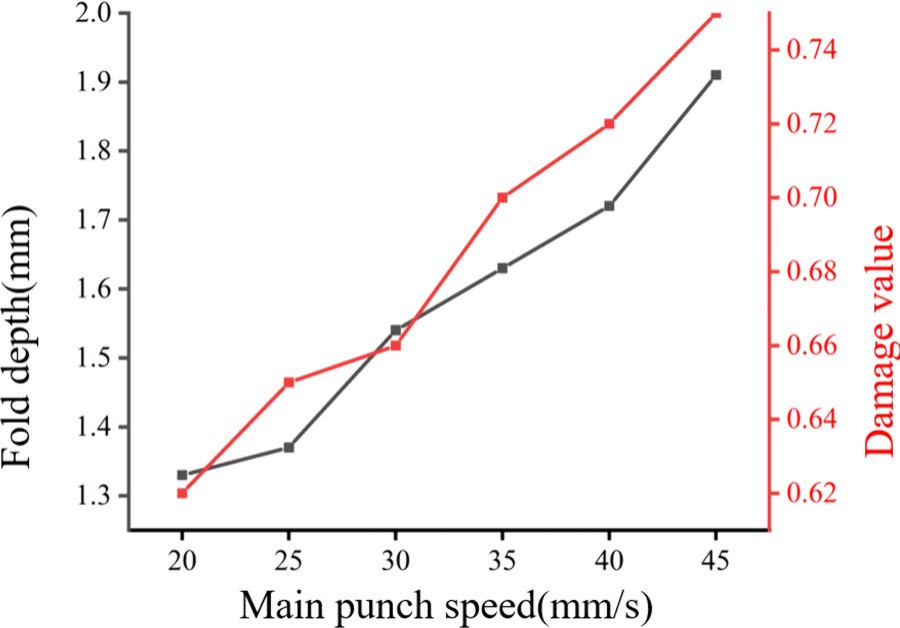
Influence of the main punch speed on the fold depth and damage value.

To quantify the influence of main punch speed on fold formation and damage accumulation, FE simulations are conducted with main punch speeds set to 20 mm/s, 25 mm/s, 30 mm/s, 35 mm/s, 40 mm/s, and 45 mm/s, respectively. All other process parameters are kept constant to isolate the effect of punch speed. The results, presented in Fig 9, demonstrate that as the main punch speed increases, both the fold depth and the damage value at the critical monitoring point P (fold apex) increase monotonically at the end of the forging process. This observation is consistent with both existing literature findings [10,28] and industrial shop-floor experience, which indicate that the operating speed of the forging press (main punch speed in this case) is a key factor influencing the occurrence of fold defects. High punch speeds can lead to uneven material flow, particularly when the die design is not optimized to accommodate such rapid deformation rates [29]. The increased flow inhomogeneity at higher punch speeds exacerbates the inward migration of surface material, thereby promoting fold formation and increasing material damage.

### 3.2 Effects of friction on velocity field evolution and damage accumulation

**Fig 10:**
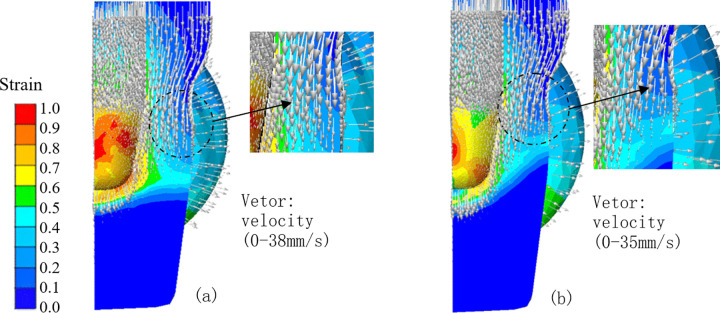
Material flow and equivalent strains at different friction conditions. (A) µ = 0, (B) µ = 0.5.

Friction at the billet-die interface plays a crucial role in determining the velocity field and damage evolution during multi-directional die forging. It exerts a significant influence on material flow within the die cavity, the formation of defects such as folds, and the accumulation of material damage [6,25,30]. To systematically investigate this effect, two extreme friction conditions: a frictionless case (µ = 0) and a high-friction case (µ = 0.5)-were simulated. The material flow velocities and equivalent strains under these two conditions are presented in Fig 10A and 10B, where velocity vectors (represented by arrows) are superimposed on equivalent strain contour plots to facilitate comprehensive analysis.

The simulation results reveal that friction significantly alters the Dead Metal Zone (DMZ)-a region of the billet that undergoes minimal or no plastic deformation-particularly in the bottom region of the billet. Under the frictionless condition (Fig 10A), the boundary between the bottom DMZ and deformed material remains approximately horizontal. Material flow vectors are vertically oriented in the circled region (as clearly shown in the magnified inset). In contrast, under the high-friction condition (Fig 10B), the boundary the bottom DMZ and the deformed material develops a steep slope, extending outward from the center toward the valve body wall. This rigid DMZ interacts with the tapered section of the die, inducing a horizontally inward material flow. This inward flow, as evidenced by the leftward orientation of velocity vectors in the circled region of Fig 10B (in contrast to the vertical orientation observed in the frictionless case), increases the probability of fold defect formation.

**Fig 11:**
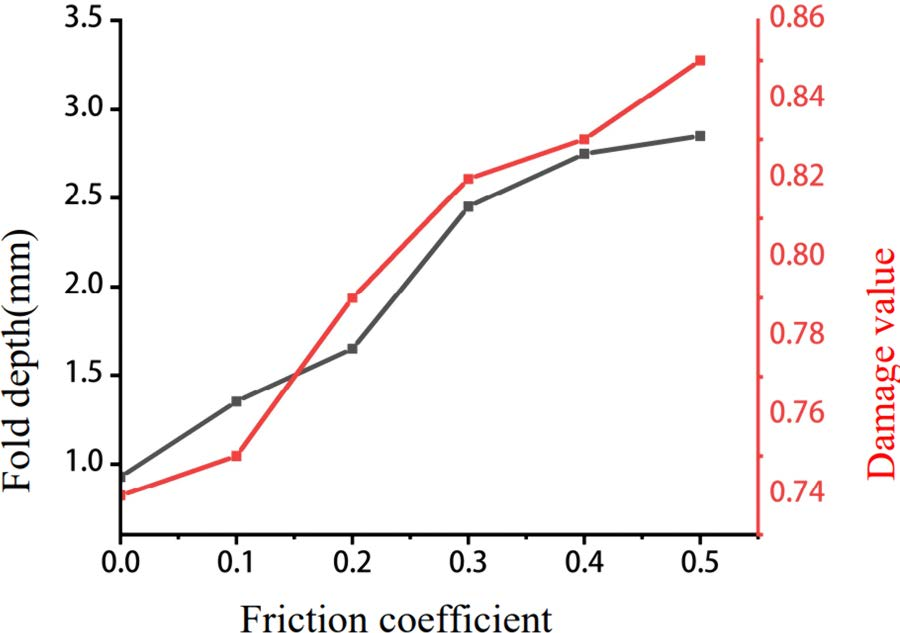
Influence of the friction coefficient on the fold depth and damage value.

To further quantify the influence of friction coefficient on fold depth and damage value, six additional FE analyses were conducted with friction coefficients set to 0, 0.1, 0.2, 0.3, 0.4, and 0.5, respectively. All other process parameters and boundary conditions are maintained constant. Fig 11 presents the fold depth and damage value at monitoring point P upon forming completion. The results confirm that an increase in the friction coefficient leads to a simultaneous increase in both fold depth and damage value—a trend that is consistent with the influence of main punch speed (Fig 9). This indicates that higher friction at the billet-die interface, like higher punch speeds, exacerbates the inhomogeneity of material flow and promotes the formation of fold defects.

### 3.3 Effects of the billet’s initial temperature on fold and damage

**Fig 12:**
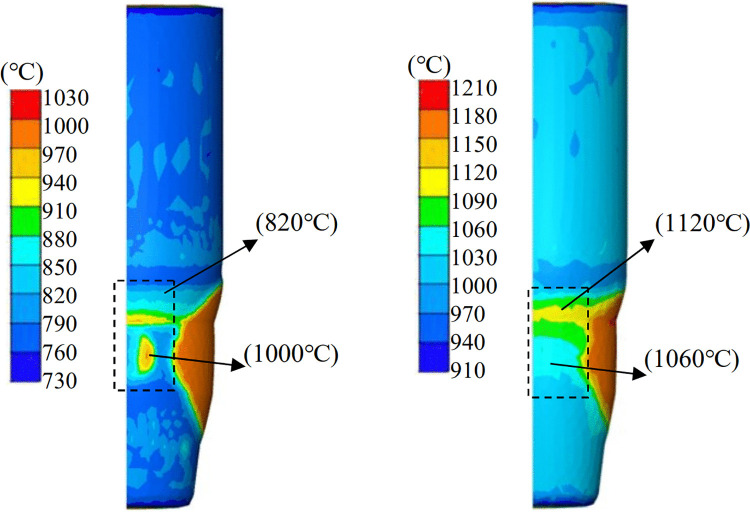
Temperature distribution with different initial temperatures of the billet. (A) 1000°C, (B) 1200°C.

Fig 12 illustrates the temperature distribution within the billet at the onset of the fold forming for two initial temperatures: 1000°C (Fig 12A) and 1200°C (Fig 12B). While both cases exhibit comparable maximum temperature differences (≈300°C) across the entire billet, significant variations are observed in the temperature gradients within the highlighted regions (boxed areas). Specifically, the internal temperature gradient within these critical regions decreases from 180°C (at an initial temperature of 1000°C) to 60°C (at an initial temperature of 1200°C), representing a reduction of 66.7%. These critical regions correspond to the initial locations of fold formation, as identified in the above simulation analyses. These regions are critical as they correspond to the initial fold formation locations. The numerical simulation results indicate that increasing the initial billet temperature significantly improves the uniformity of its temperature field. Inhomogeneous temperature distribution results in inhomogeneous deformation and uneven material flow [31]. The reduction in temperature gradient achieved by increasing the initial billet temperature helps to mitigate material flow inhomogeneity by alleviating the differences in plastic flow behavior induced by temperature variations [32–34].

**Fig 13:**
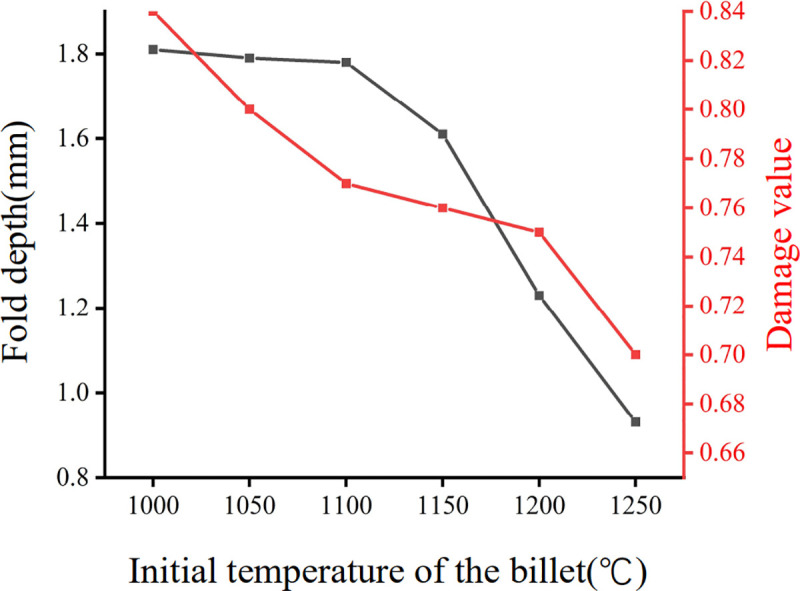
Influence of the initial billet temperature on the fold depth and damage value.

To systematically evaluate the effect of billet initial temperature on fold formation and damage accumulation, FE simulations were conducted with initial billet temperatures set to 1000°C, 1050°C, 1150°C, 1200°C, and 1250°C, respectively. All other process parameters are kept constant to isolate the temperature effect. The results, presented in Fig 13, demonstrate that increasing the initial billet temperature effectively reduces fold defects and material damage.

The numerical simulations conducted in Section 2 have visually demonstrated the fold formation process, clearly identifying the inward flow of surface material as the root cause of fold defects. The analyses in Sections 3.1 and 3.2 further indicate that both high main punch speeds and high friction coefficients contribute to increased inward material flow velocities, thereby facilitating the inward migration of surface material and promoting fold formation. Specifically, elevated friction coefficients at the billet-die interface induce localized shear stresses, which disrupt the uniform material flow pattern. This phenomenon is particularly pronounced in regions with complex cross-sectional geometries (such as the tapered section of the valve body), where differential flow velocities across the billet-die contact interface create material accumulation zones [35]. High friction conditions exacerbate strain localization, causing outer material layers to override inner sections during deformation–a primary mechanism for fold initiation.

Moreover, generally speaking, under high-temperature conditions, the material’s plastic deformation capability is enhanced [36,37], leading to more uniform material flow within the die cavity, thereby effectively alleviating localized stress concentration. This homogeneous flow facilitates complete filling of the die cavity, preventing local material accumulation or fold defects caused by flow obstruction. Furthermore, elevated temperatures may reduce the friction coefficient at the billet-die interface, improving interfacial sliding behavior and further minimizing flow inhomogeneity, fold, or surface damage induced by frictional resistance.

### 3.4 Orthogonal experiments

#### 3.4.1 Factor selection for orthogonal experiment.

In die forging processes, die speed, billet initial temperature, and billet-die friction coefficient are widely recognized as key influencing factors. This recognition is based on their significant impacts on metal flow behavior, deformation uniformity, and the formation of defects such as folds [28,38,39]. The friction condition at the die-billet interface governs the shear resistance and thus influences the metal flow pattern. Excessive friction restricts surface metal movement, leading to internal metal overrunning the front layer, which can result in backflow or fold defects. Initial billet temperature determines the material’s ductility and flow stress. Lower temperatures increase deformation resistance and reduce plasticity, making it difficult for metal to fill intricate cavities completely. Conversely, higher temperatures enhance formability by promoting dynamic recrystallization and reducing yield strength, thereby minimizing the risk of fold defects. Die speed (or punch speed) affects the strain rate experienced by the material and thermomechanical coupling during forging. High punch speeds may lead to excessive adiabatic heating and thermal gradients, while low speeds can cause significant heat loss, cooling the leading edge of the billet. When subsequent metal pushes against this cooled front, folding is likely to occur. Therefore, these three factors are strategically chosen because they are both practically adjustable and scientifically meaningful in finite element analysis and in industrial trials, allowing researchers to systematically optimize process parameters and improve forging quality.

#### 3.4.2 Orthogonal experimental design.

The core principle of orthogonal experimental design lies in the use of orthogonal arrays—standardized, balanced test plans—to efficiently screen the key influencing factors (and their interaction effects) on the experimental results, while minimizing the total number of experimental runs. This design methodology ensures two critical characteristics: first, each factor’s levels are tested evenly, avoiding bias caused by over-testing or under-testing specific levels; second, the combination of any two factors’ levels is equally represented in the experimental plan. This balanced design makes the experimental results statistically analyzable, enabling the isolation of the individual impact of each factor on the response variables (fold depth and damage value). By striking a balance between “test efficiency” and “result reliability”, orthogonal experimental design has become widely used in the optimization of manufacturing processes, product development, and scientific experiments [40].

The primary advantage of orthogonal experiments lies in their efficiency. By systematically varying factors and levels, researchers can obtain comprehensive insights with fewer experimental runs compared to full factorial designs. This efficiency is particularly beneficial in industrial applications where time and resources are limited [35,41,42]. For the three selected process parameters, an orthogonal experimental design is employed to optimize the valve-body forging process. Each parameter is assigned three levels, which results in a three-level, three-factor orthogonal experiment. In orthogonal array design, the number of levels per factor must match the number of levels specified for that factor in the experiment, while the number of factors can be less than or equal to the number of the array’s column count [43,44]. Therefore, the standard L9(3^4^) orthogonal array was adopted for the experimental design, which is well-suited for three-factor, three-level experiments.

The procedure for the orthogonal experimental design involves two key steps: the calculation of mean response values and range analysis.

First, the mean response values (fold depth or damage value) $k_{i}$ for fold depth and damage value at each level of the three factors are calculated using equation (2):


$$\begin{array}{c} k_{i}=\frac{\text{1}}{n}\sum {\mathrm{\backslash nolimits}}_{j=\text{1}}^{n}Y_{ij},\;(i=\text{1},..,\text{6},\;j=\text{1},\text{2},\text{3},\;n=\text{3}) \end{array}$$ (2)

where $Y_{ij}$ represents the folding depth or damage value under the *j*-th level of the *i*-th factor, n=3, the number of the experiment conducted under the *j*-th level of the *i*-th factor.

Second, range analysis is performed to evaluate the relative importance of each factor on the response variables. The range values for fold depth ($R_{\text{1}}$) and damage value ($R_{\text{2}}$) are calculated using equations (3) and (4),


$$R_{\text{1}}=max\left(k_{\text{1}},k_{\text{2}},k_{\text{3}}\right)-min\left(k_{\text{1}},k_{\text{2}},k_{\text{3}}\right)$$ (3)

$$R_{\text{2}}=max\left(k_{\text{4}},k_{\text{5}},k_{\text{6}}\right)-min\left(k_{\text{4}},k_{\text{5}},k_{\text{6}}\right)$$ (4)

where $R_{\text{1}}$, $R_{\text{2}}$ are the ranges of the fold depth and damage value, respectively. $k_{\text{1}},k_{\text{2}},k_{\text{3}}$ are the means of the fold depth, $k_{\text{4}},k_{\text{5}},k_{\text{6}}$, the damage value. A larger range indicates a greater spread in possible values, suggesting a more significant influence of that factor on the outcome.

The detailed orthogonal experimental design and the corresponding results are presented in Table 4. Based on the analysis of range values, it can be concluded that friction has the most significant impact on the fold depth and damage value, while the billet’s initial temperature exerts a greater influence on the fold and damage than the main punch speed. Therefore, the optimized process parameter combination can be determined as: a friction factor of 0.1, the billet’s initial temperature of 1200°C, the main punch speed of 30 mm/s.

**Table 4:** Orthogonal array and results.

Column No.	1	2	3	–	–
Factor	Initial billet temperature (°C)	Main punch speed (mm/s)	Coefficient of friction	Fold depth (mm)	Damage value
experiment1	1100	20	0	3.65	0.738
experiment2	1100	30	0.1	2.70	0.580
experiment3	1100	40	0.2	3.45	0.701
experiment4	1150	20	0.1	2.85	0.595
experiment5	1150	30	0.2	3.05	0.655
experiment6	1150	40	0	3.55	0.598
experiment7	1200	20	0.2	2.95	0.672
experiment8	1200	30	0	2.85	0.570
experiment9	1200	40	0.1	2.50	0.443
K1	3.2667	3.1500	3.3500	–	–
K2	3.1500	2.8667	2.6833	–	–
K3	2.7667	3.1667	3.1500	–	–
$R_{\text{1}}$	0.5010	0.3000	0.6667	–	–
K4	0.6730	0.6683	0.6353	–	–
K5	0.6160	0.5807	0.5393	–	–
K6	0.5617	0.6017	0.6760	–	–
$R_{\text{2}}$	0.1113	0.0876	0.1367	–	–

#### 3.4.3 Statistical significance of each factor on the response variables.

Analysis of variance (ANOVA) is a statistical test used to compare the means of multiple groups, enabling the quantification of the statistical significance of each factor’s influence on the response variables. The calculation of ANOVA adheres to the standard procedure, and the mathematical formulas are briefly outlined as follows:

1) Total sum of squares ($ss_{T}$):this represents the total variation in the response variable, calculated as:

$$\begin{array}{c} ss_{T}=\sum_{i=\text{1}}^{n}{\left(y_{i}-\overset{―}{y}\right)}^{\text{2}} \end{array}$$ (5)

where $y_{i}$ is the measured value, $\overset{―}{y}$, the overall mean, and *n*, the total number of experiments.

2) Sum of squares for a factor $SS_{F}$: this represents the variation in the response variable attributed to a specific factor. For factors *A* (billet’s initial temperature), *B* (main punch speed), and *C* (friction coefficient), the sum of squares is calculated as:

$$\begin{array}{c} SS_{A}=\sum_{j=\text{1}}^{a}n_{j}{\left({\overset{―}{y}}_{Aj}-\overset{―}{y}\right)}^{\text{2}}\\ SS_{B}=\sum_{j=\text{1}}^{b}n_{j}{\left({\overset{―}{y}}_{Bj}-\overset{―}{y}\right)}^{\text{2}}\\ SS_{C}=\sum_{j=\text{1}}^{c}n_{j}{\left({\overset{―}{y}}_{Cj}-\overset{―}{y}\right)}^{\text{2}} \end{array}$$ (6)

where a, b, c are the number of levels of factors A, B, and C, respectively; ${\overset{―}{y}}_{Aj},\;{\overset{―}{y}}_{Bj},\;{\overset{―}{y}}_{Cj}$ are the mean values of the response variable at each level of factors *A*, *B*, and *C*, respectively; and $n_{j}$is the number of observations at that level.

3) Error (residual) sum of squares ($SS_{E}$): this represents the variation in the response variable attributed to random errors, calculated as:

$$\begin{array}{c} SS_{E}=SS_{T}-\left(SS_{A}+SS_{B}+SS_{C}\right) \end{array}$$ (7)

4) Mean square:

$$\begin{array}{c} MS_{A}=\frac{SS_{A}}{{df}_{A}},\;MS_{B}=\frac{SS_{B}}{{df}_{B}},\;MS_{A}=\frac{SS_{C}}{{df}_{c}} \end{array}$$ (8)

$$\begin{array}{c} MS_{E}=\frac{SS_{E}}{{df}_{E}} \end{array}$$ (9)

where ${df}_{A},\;{df}_{B},{df}_{C}$ and ${df}_{E}$ are the degrees of freedom for factors *A*, *B*, *C* and the error, respectively.

5) F-statistic: this is the ratio of the mean square of a factor to the mean square of the error, used to test the significance of the factor’s influence:

$$\begin{array}{c} F=\frac{MS_{A}}{MS_{E}},\;F=\frac{MS_{B}}{MS_{E}},\;F=\frac{MS_{C}}{MS_{E}}. \end{array}$$ (10)

A higher F-value implies a more substantial impact of the corresponding factor on the outcome. When the calculated F-value exceeds a critical F-value ($F_{crit}$) obtained from a statistical table, the factor is considered to have a statistically significant influence on the response variable [45].

6) P-value:

$$\begin{array}{c} P=\frac{\text{1}}{F+\text{1}} \end{array}$$ (11)

A smaller p-value indicates a lower likelihood that the experimental results are caused by random error, implying that the factor has a more significant influence on the response variable [46]. For the purposes of this study, a p-value less than 0.05 is considered statistically significant.

ANOVA tests were conducted to assess the effect of the three factors: billet’s initial temperature, main punch speed, and friction coefficient on the fold depth and damage value, respectively. The results for fold depth are presented in Table 5, and those for damage value are shown in Table 6.

**Table 5:** ANOVA test results on the fold depth.

Factor	Degrees of freedom	Sum of squares	Mean square	F-value	P-value	Significance
Billet temperature	2	0.4101	0.2051	186.4545	0.0053	Significant
Main punch speed	2	0.1686	0.0843	153.2727	0.0064	Significant
Friction coefficient	2	0.7020	0.3510	319.0909	0.0031	Significant
Error	2	0.0021	0.0011	–	–	–

**Table 6:** ANOVA test results on the damage value.

Factor	Degrees of freedom	Sum of squares	Mean square	F-value	P-value	Significance
Billet temperature	2	0.0185	0.0093	46.2500	0.0212	Significant
Main punch speed	2	0.0124	0.0062	31.0000	0.0313	Significant
Friction coefficient	2	0.0296	0.0148	98.6667	0.0100	Significant
Error	2	0.0003	0.0002	–	–	–

For fold depth (Table 5) the F-values corresponding to the friction coefficient, billet’s initial temperature, and main punch speed are 319.0909, 186.4545, and 153.2727, respectively. These values are significantly higher than the critical F-value ($F_{crit}=\text{1}\text{9}$ [47]), and the corresponding P-values are less than 0.05. This fully demonstrates that these three factors have a significant influence on the fold depth. Moreover, the order of these F-values-friction coefficient>billet’s initial temperature>main punch speed- also indicates the relative degree of influence of their corresponding factors on the fold depth. Specifically, the friction coefficient has the greatest influence on the fold depth, followed by the billet’s initial temperature, and the main punch speed has the least influence. With respect to the damage value (Table 6), it can readily be observed that the results follow the same pattern: the friction coefficient exhibits the highest F-value (98.6667) and the smallest p-value (0.0100), followed by the billet initial temperature (F = 46.2500, p = 0.0212) and the main punch speed (F = 31.0000, p = 0.0313).. Notably, these results are consistent with the range analysis results above, further proving the reliability and validity of the experimental results.

### 3.5 FE simulation with optimized process parameters

**Fig 14:**
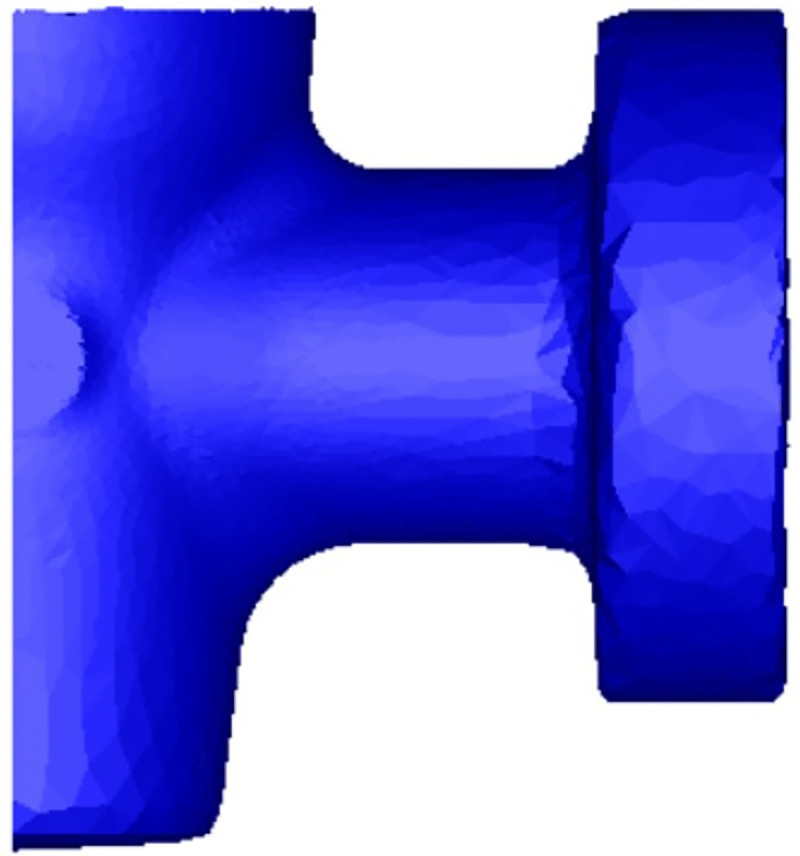
Simulated valve body forging with optimized process parameters.

**Fig 15:**
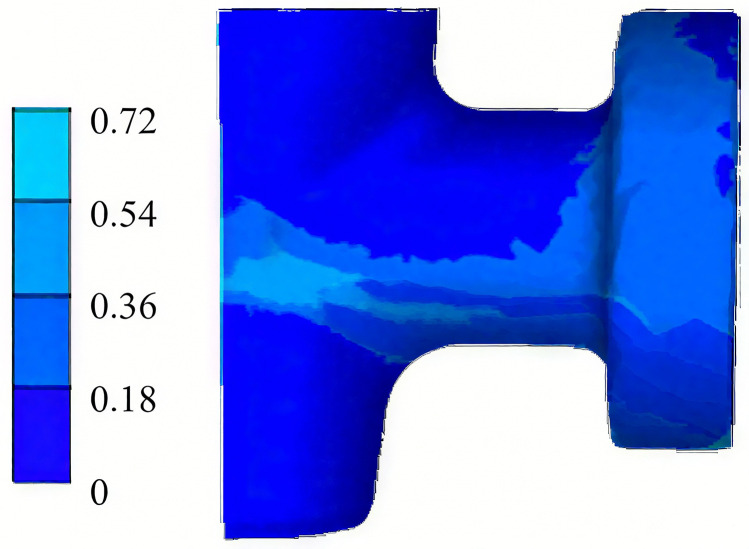
Damage value with optimized process parameters.

To validate the effectiveness of the optimized process parameters, an FE simulation is conducted using the determined optimal combination: a friction coefficient of 0.1, a billet initial temperature of 1200°C, and a main punch speed of 30 mm/s. The results, presented in Fig 14, demonstrate that the optimized process can produce a valve body forging with well-defined contours and defect-free surfaces. This indicates that the optimized parameters effectively suppress the formation of fold defects. Furthermore, as shown in Fig 15, the damage values under the optimized parameters are significantly reduced compared to those observed in the unoptimized process (Fig 7). This reduction in damage values indicates a substantial improvement in the forging quality, confirming a lower risk of crack initiation in the final forged component.

## 4. Production verification

**Fig 16:**
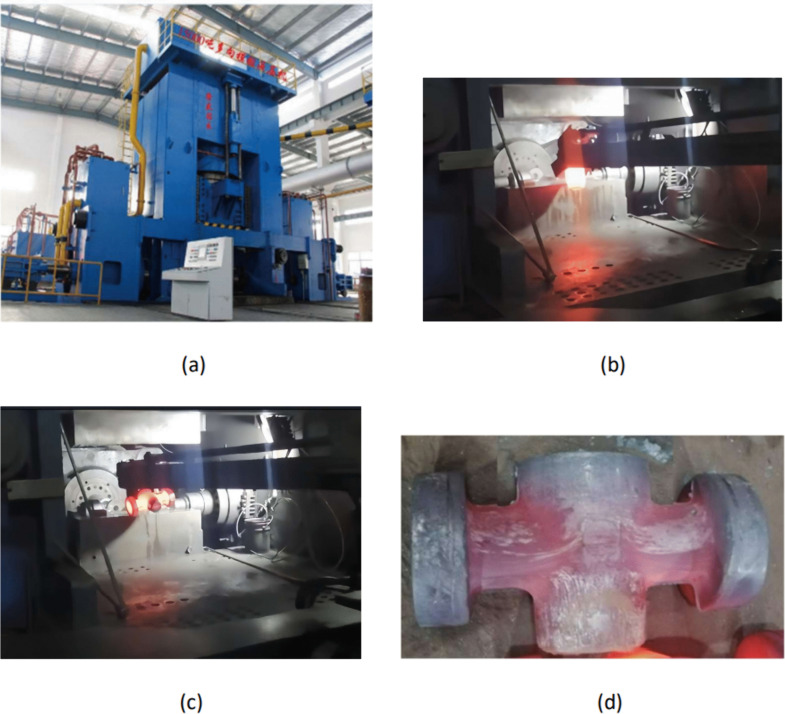
(A) 14,500-ton multi-ram press (B) billet being positioned into the die cavity (C) removing the forged valve body from the die cavity (D) the forged valve body.

To further validate the industrial applicability of the optimized process parameters, production verification was conducted using a 14,500-ton multi-ram forging press at Jiangsu Longsheng Drilling Machinery Manufacturing Co., Ltd. (Fig 16A). The production process follows a well-defined sequence: first, the dies are lubricated with water-based graphite to reduce friction at the billet-die interface, and the billet is subjected to descaling to remove surface oxide layers. The preformed billet, heated to the optimized initial temperature of 1200°C, is then placed into the lower die cavity from the front (Fig 16B). Next, the upper die is lowered to engage with the lower die, forming a closed cavity with only an opening reserved for the rear punch to pass through. Once the rear punch advances to its designated position to form the main channel of the valve body, the left and right punches are activated and advance to their operational positions to form the side ports. Finally, the upper die is lifted, and the forged valve body is removed from the die cavity (Fig 16C). Fig 16D presents the final multi-directional die forged valve body, which exhibits excellent surface quality and dimensional conformity to the design requirements.

As illustrated in Fig 16B, after lubricating the dies with water-based graphite and descaling the billet, the preformed billet heated to 1200°C was placed into the lower die cavity from the front. Subsequently, the upper die was lowered to engage with the lower die to form a closed cavity, retaining only an opening for the rear punch to pass through. Upon the rear punch advancing to its designated position to form the valve body’s main channel, the left and right punches advanced to their operational positions. Finally, operators lifted the upper die and removed the forging from the die cavity (Fig 16C). Fig 16D presents a multi-directional die forged valve body exhibiting excellent surface quality and dimensional conformity to the design requirements.

**Fig 17:**
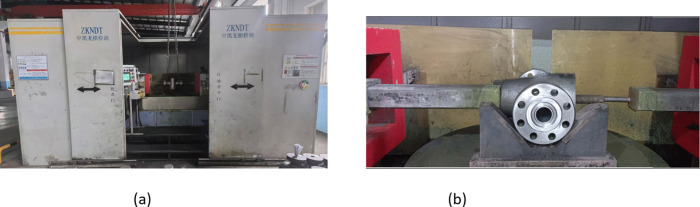
(A) CJW-10000 Fluorescent Magnetic Particle Flaw Detector (B) Forged part to be tested.

To ensure the absence of surface and near-surface defects (such as folds and cracks) in the forged valve bodies, Magnetic Particle Inspection (MPI)—a non-destructive testing (NDT) method—was performed. The MPI was conducted using a fluorescent wet technique with a CJW-10000 Fluorescent Magnetic Particle Flaw Detector (a valve-body specialized model, shown in Fig 17). The key test parameters are documented in Table 7, which are set in accordance with the ASTM E709 standard to ensure the reliability and accuracy of the inspection results. The UV-A illumination intensity was measured as 7826 μW/cm² before use and 8468 μW/cm² after use (a requirement for battery-powered UV lights), which meets the minimum intensity requirements specified in the standard.

**Table 7:** Test parameters for the fluorescent magnetic particle flaw detection.

Parameters	Value
Magnetizing time	3s
Circumferential magnetizing current	3000A
Longitudinal magnetizing current	2500A
Bath strength	0.2 ml/ 100 ml
White light intensity	18lx

**Fig 18:**
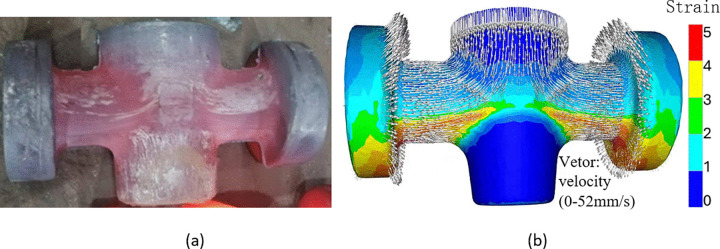
Comparison between, (A) real product and (B) simulated valve.

Fig 18A depicts the multi-directional die-forged valve body, while Fig 18B presents the corresponding FE simulation. In the simulation results, the contour map illustrates the equivalent strain distribution and the arrows denote material flow velocity (ranging from 0 to 52 mm/s). Notably, the experimental and simulated shapes of the valve body exhibit perfect agreement, confirming the accuracy of the FE model.

Distinct streaks, induced by severe plastic deformation and rapid material flow during the high-energy multi-directional die forging process, are clearly visible on the actual valve body. These streaks are consistent with the material flow patterns predicted by the FE simulation, further validating the model’s ability to capture the complex metal flow dynamics.

**Fig 19:**
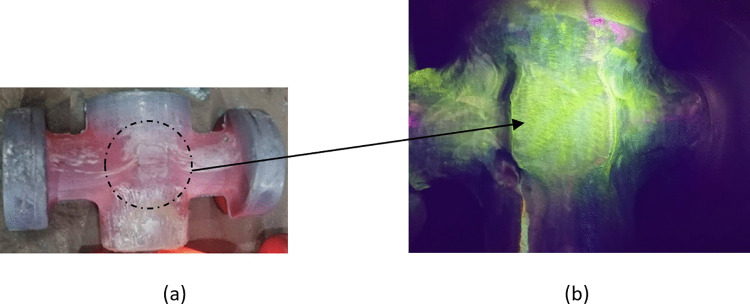
MPI result. (A) multi-directional forged valve body (B) homogeneous magnetic particle dispersion.

Fig 19 presents the MPI results: Fig 19A shows the multi-directional die forged valve body to be inspected, and Fig 19B is a close-up view of the valve body surface under UV illumination. The results reveal a homogeneous fluorescent magnetic particle dispersion across the forging surface, with no evidence of particle accumulation that is a key indicator of surface or near-surface discontinuities (such as folds or cracks). The absence of such accumulation confirms that the forged valve bodies are free from detectable defects, complying with the acceptance criteria specified in the ASTM E709 standard. This result not only validates the integrity of the forged component but also underscores the effectiveness of the optimized multi-directional die forging process in minimizing surface and sub-surface defects.

## 5. Challenges in practical implementation

In addition to the primary factors discussed above, the successful implementation and long-term robustness of the fold prevention methodology developed in this study may be further influenced by some other process-related variables inherent in the industrial forging environment. These variables, although often overlooked in laboratory-scale simulations, can have a significant impact on the quality and consistency of the forged products in practical production.

1) Non-uniform billet heating: it leads to spatial variations in flow stress during deformation [48]. Regions of the billet with higher temperatures exhibit lower yield strength and greater plasticity, which can cause localized metal over-flow. In contrast cooler regions of the billet resist deformation, resulting in strain concentration and potential defects such as folding or cracking.
2) Presence of surface scale (oxide layer): the oxide scale formed on the billet surface during heating profoundly affects heat transfer efficiency and friction characteristics at the die-billet interface. Oxide layers act as thermal barriers, reducing the heat transfer coefficient between the forging and the die. Moreover, scale particles can become embedded in the workpiece surface or abrade the die, contributing to surface defects and accelerated wear.
3) Machine tool rigidity: it governs the system’s ability to maintain precise die alignment and closure under high forming loads, thereby affecting overall process stability and part consistency. Insufficient rigidity results in elastic deflection of frames, misalignment of dies, and incomplete filling of cavities, particularly in precision die forging operations.
4) Progressive die wear: it alters the geometry of the die cavity over successive forging cycles, leading to dimensional inaccuracies, poor surface finish, and even failure to fill critical features of the final product. In practical production, monitoring and compensating for die wear are essential to maintain the consistency of the forging process and prevent the recurrence of fold defects.

These secondary yet critical parameters collectively modulate the thermo-mechanical response at the die-billet interface, influencing metal flow patterns, stress distribution, and defect evolution—particularly fold formation mechanisms. Therefore, the real-world applicability of the proposed methodology must account for such process variabilities. To ensure reliable performance across diverse production conditions, it is recommended that industrial users implement additional process control measures, such as real-time temperature monitoring of the billet, advanced descaling techniques, regular maintenance of the forging press to ensure rigidity, and periodic inspection and repair of the dies to mitigate the effects of wear.

## 6. Conclusions

1) The mechanism of fold defect formation is clarified: In the multi-directional die forging of valve bodies, the fundamental cause of fold defects is the inward flow of material on the billet surface. The tapered section of the die changes the material flow velocity from being mainly vertically downward to downward-leftward, and the surface material flows inward at a faster rate, leading to material accumulation.
2) Univariate analysis of fold-influencing factors revealed that: higher initial billet temperature, lower friction coefficient, or slower main punch speed help reduce the fold depth. The spatial overlap between the fold region and the region with high damage values suggests a significant likelihood of crack initiation.
3) Orthogonal experiment and ANOVA test results demonstrated that friction coefficient exhibits statistically dominant effects on both fold formation and material damage; initial billet temperature imposes greater influence than main punch speed on fold depth and damage accumulation; the optimal parameter combination for defect-free forming is: friction coefficient, 0.1, initial billet temperature, 1200°C, and main punch speed, 30 mm/s.
4) Production trials and magnetic particle inspection (MPI) verify that with the optimized parameters, multi-directional die forging can mass produce qualified valve bodies exhibiting excellent surface quality and dimensional conformity.
5) Secondary variables should be considered in practical application: In addition to the aforementioned key parameters, secondary factors such as uneven billet heating, surface oxide scale, insufficient machine tool rigidity, and die wear may also affect the control of fold defects. In actual production, measures such as precise temperature control, billet descaling, equipment maintenance, and regular die inspection should be adopted to further ensure the stability of forging quality and guarantee the reliable application of the optimized process under different production conditions.

## Supporting information

S1 File Data supplied for the manuscript. (DOCX)

S2 File Data for Fig 6. (XLSX)
