# Supplementary material for: Study on fold formation mechanism and process optimization in multi-directional die forged valve bodies
Source: PLoS One. 2025 Dec 26;20(12):e0337844. doi: 10.1371/journal.pone.0337844 (PMC12742751; doi:10.1371/journal.pone.0337844)
Supplement: S1 File — (DOCX) [file pone.0337844.s001.docx]

Data supplied for the manuscript

1. Data used in Figure 3

| **Data point number** | **1** | **2** | **3** | **4** | **5** | **6** |
| --- | --- | --- | --- | --- | --- | --- |
| **Number elements of billet’s initial mesh** | 8313 | 15154 | 29157 | 38565 | 50375 | 57543 |
| **Temperature (℃）** | 1080.16 | 1077.59 | 1077.21 | 1078.93 | 1072.53 | 1077.55 |
| **Strain value** | 4.588 | 4.377 | 4.720 | 4.767 | 4.515 | 4.634 |
| **Computation time(min）** | 17 | 25 | 46 | 80 | 113 | 133 |

1. Data used in Figure6 (Double click the table below to open it. If it doesn’t work, check the attached Excel file titled with DataForFigure6)

1. Data used in Figure9

| **Main punch speed(mm/s）** | **20** | **25** | **30** | **35** | **40** | **45** |
| --- | --- | --- | --- | --- | --- | --- |
| **Fold depth (mm)** | 1.33 | 1.37 | 1.54 | 1.63 | 1.72 | 1.91 |
| **Damage value** | 0.62 | 0.65 | 0.66 | 0.70 | 0.72 | 0.75 |

1. Data used in Figure11

| **Friction coefficient** | **0** | **0.1** | **0.2** | **0.3** | **0.4** | **0.5** |
| --- | --- | --- | --- | --- | --- | --- |
| **Fold depth (mm)** | 0.93 | 1.35 | 1.65 | 2.45 | 2.75 | 2.85 |
| **Damage value** | 0.74 | 0.75 | 0.79 | 0.82 | 0.83 | 0.85 |

1. Data used in Figure13

| **Initial temperature of the billet(℃）** | **1000** | **1050** | **1100** | **1150** | **1200** | **1250** |
| --- | --- | --- | --- | --- | --- | --- |
| **Fold depth (mm)** | 0.93 | 1.23 | 1.61 | 1.78 | 1.79 | 1.81 |
| **Damage value** | 0.7 | 0.75 | 0.76 | 0.77 | 0.8 | 0.84 |

1. Relevant calculation for Table4

| K11=(3.65 + 2.70 + 3.45)/3 = **3.2667** | K13=(3.65 + 3.55 + 2.85)/3 = **3.3500** |
| --- | --- |
| K21=(2.85 + 3.05 + 3.55)/3 = **3.1500** | K23=(2.70 + 2.85 + 2.50)/3 = **2.6833** |
| K31=(2.95 + 2.85 + 2.50)/3 = **2.7667** | K33=(3.45 + 3.05 + 2.95)/3 = **3.1500** |
| K12=(3.65 + 2.85 + 2.95)/3 = **3.150**0 | R11=3.27−2.77=0.5010 |
| K22=(2.70 + 3.05 + 2.85)/3 = **2.8667** | R12=3.17−2.87=0.3000 |
| K32=(3.45 + 3.55 + 2.50)/3 = **3.1667** | R13=3.35−2.68=0.6667 |

| K41=(0.738 + 0.580 + 0.701)/3 = **0.6730** | K43=(0.738 + 0.598 + 0.570)/3 = **0.6353** |
| --- | --- |
| K51=(0.595 + 0.655 + 0.598)/3 = **0.6160** | K53=(0.580 + 0.595 + 0.443)/3 = **0.5393** |
| K61=(0.672 + 0.570 + 0.443)/3 = **0.5617** | K63=(0.701 + 0.655 + 0.672)/3 = **0.6760** |
| K42=(0.738 + 0.595 + 0.672)/3 = **0.6683** | R21=0.668 − 0.580 = **0.1113** |
| K62=(0.701 + 0.598 + 0.443)/3 = **0.5807** | R22=0.668 − 0.580 = **0.0876** |
| K52=(0.580 + 0.655 + 0.570)/3 = **0.6017** | R33=0.676 − 0.539 = **0.1367** |

Relevant calculation for Table5 and Table 6

$\bar{y}$= [3.65+2.70+3.45+2.85+3.05+3.55+2.95+2.85+2.50]/9=3.0611

SS_total_= (3.65-3.0611)^2^+(2.70-3.0611)^2^+(3.45-3.0611)^2^+(2.85-3.0611)^2^+(3.05-3.0611)^2^+(3.55-3.0611)^2^+(2.95-3.0611)^2^+(2.85-3.0611)^2^+(2.50-3.0611)^2^ =20.3468+0.1304+0.1512+0.0446+0.0001+0.2390+0.0123+0.0446+0.3148=1.2828

SS_tempture_=3*[(3.2667-3.0611)^2^+(3.15-3.0611)^2^+(2.7667-3.0611)^2^]

=0.0422+0.0079+0.0866=0.4101

SS_speed_=3*[(3.1500-3.0611)^2^+(2.8667-3.0611)^2^+(3.1667-3.0611)^2^]

=0.0079+0.0377+0.0111=0.1686

SS_friction_=3*[(3.3500-3.0611)^2^+(2.6833-3.0611)^2^+(3.1500-3.0611)^2^]

=0.0834+0.1427+0.0079=0.7020

SS_E_=1.2828-0.4101-0.1686-0.7020=0.0021

${df}_{total}$=N−1=9−1=8

${df}_{A}$_=_${df}_{B}={df}_{C}$ =3−1=2

${df}_{E}$=8−6=2

MS_t_=0.4101/2=0.2051

MS_s_=0.1686/2=0.0843

MS_f_=0.7020/2=0.3510

MS_E_=0.0021/2=0.0011

F_t_=0.2051/0.0011=186.4545

F_s_=0.1686/0.0011=153.2727

F_f_=0.7020/0.0011=319.0909

P_t_=1/（1+186.4545）=0.0053

P_s_=1/（1+153.2727）=0.0064

P_f_=1/（1+319.0909）=0.0031

Similarly, the critical F-value for **Table 6** can be determined in the same way.

$\bar{y}$=0.6169

SS_total_ =0.0147+0.0014+0.0071+0.0003+0.0015+0.0004+0.0030+0.0022+0.0302=0.0608

SS_tempture_=0.0094+0+0.0091=0.0185

SS_speed_ =0.0079+0.0007+0.0039=0.0124

SS_friction_ =0.0010+0.0181+0.0105=0.0296

SS_E_=0.0003

${df}_{total}$ =N−1=9−1=8

${df}_{A}$_=_${df}_{B}={df}_{C}$ =3−1=2

${df}_{E}$ =8−6=2

MS_t_=0.0093

MS_s_=0.0062

MS_f_=0.0148

MS_E_=0.0002

Ft=46.2500

Fs=31.0000

F_f_=98.6667

F_t_=46.2500

F_s_=31.0000

F_f_=98.6667

P_t_=1/（1+46.2500）=0.0212

P_s_=1/（1+31.0000）=0.0313

P_f_=1/（1+98.6667）=0.0100
